# Influence of Zeolite Coating on the Corrosion Resistance of AZ91D Magnesium Alloy

**DOI:** 10.3390/ma7086092

**Published:** 2014-08-22

**Authors:** P. Chakraborty Banerjee, Ren Ping Woo, Sam Matthew Grayson, Amrita Majumder, R. K. Singh Raman

**Affiliations:** 1 Departments of Mechanical and Aerospace Engineering, Monash University, Melbourne, VIC 3800, Australia; E-Mail: raman.singh@monash.edu; 2 Department of Chemical Engineering, Monash University, Melbourne, VIC 3800, Australia; E-Mails: rpwoo1@student.monash.edu (R.P.W.); smgra10@student.monash.edu (S.M.G.); amrita.majumder@monash.edu (A.M.)

**Keywords:** magnesium alloys, zeolite coating, electrochemical impedance spectroscopy, corrosion, AZ91

## Abstract

The protective performance of zeolite coating on AZ91D magnesium alloy was evaluated using potentiodynamic polarisation and electrochemical impedance spectroscopy (EIS) in 0.1 M sodium chloride solution (NaCl). Electrical equivalent circuit (EEC) was developed based upon hypothetical corrosion mechanisms and simulated to correspond to the experimental data. The morphology and the chemical nature of the coating were characterized by scanning electron microscopy (SEM) and X-ray diffraction (XRD) analysis. Post corrosion morphologies of the zeolite coated and the uncoated AZ91D alloy were investigated using SEM. The corrosion resistance of the zeolite coated specimen was at least one order of magnitude higher than the uncoated specimen.

## 1. Introduction

Magnesium and its alloys are the lightest structural material and hence, are very attractive for the automotive and aerospace industries. However, they suffer from severe corrosion, which is generally attributed to the low standard reduction potential of magnesium and its ability to form only a partially protective surface film [1]. Other factors that generally facilitate corrosion are certain impurities (such as, iron (Fe), copper (Cu), Nickel (Ni)) commonly found in magnesium alloys and the micro-galvanic coupling between the intermetallic particles and the α-solid solution [1]. Different approaches have been investigated to improve the corrosion resistance of magnesium alloys, e.g., anodizing, plating, polymer coatings, conversion coatings and so on.

Zeolite coatings are emerging as an alternative environmentally friendly coating for metallic substrates. Zeolites are a class of micro porous crystalline aluminosilicate based on a 3D network of oxygen ions. Zeolites are constructed from AlO_4_ and SiO_4_ tetrahedra linked to each other by sharing oxygen ions. The empirical formula of zeolite is, *m*_2/*n*_·Al_2_O_3_·*x*SiO_2_·*y*H_2_O, where *m* is an exchangeable cation, *n* is the valence of the cation, *x* is ≥2 (as Al^3+^ does not occupy the adjacent tetrahedral) and *y* is the degree of hydration [2]. In order to effectively use zeolite as a barrier coating, it is essential to eliminate the porosities of the zeolite structure by a good intergrowth [3,4,5] (to eliminate intercrystal porosity) and by blocking the pores with structure directing agent (SDA) molecules [6] (to eliminate the intracrystal porosities). Bulky molecules (such as, tetrapropylammonium molecules, TPA) are generally used as SDAs for synthesis of high silica ZSM-5 zeolite systems and are left trapped inside the crystal. A space filling model suggested that ZSM-5 has a channel size of 5.5 Å and TPA molecules tightly fit in the channels [6]. These trapped SDAs are generally stable at high temperatures (≥350 °C) in the presence of oxygen [3,4,5,7]. Zeolite films synthesized using SDAs are reported to be impermeable to gases, as result of low levels of both inter and intracrystal porosities [3,4,5].

Owing to its inert nature and impermeability, zeolite coatings have been used as a barrier coating for steel, aluminium alloys and a magnesium alloy [8,9,10,11,12,13,14,15]. Cheng *et al.* [8] and Beving *et al.* [10] had synthesized zeolite coating on various aluminium alloys (AA2024-T3, AA5052-H32, AA6061-T4 and AA7075-T6) by *in situ* crystallization method. They reported that *in situ* crystallization method developed uniform and dense zeolite coatings, which significantly improved the corrosion resistance of these aluminium alloys in various acidic, neutral and basic electrolytes. McDonnel *et al.* [9] synthesized zeolite coating by *in situ* crystallization on 304 stainless steel used as condensers in spacecraft and have reported the antimicrobial efficacy of the system. Another method of synthesis is the incorporation of zeolite powders in a sol-gel matrix. Dias *et al.* [13] and Calabrese *et al.* [12] have synthesized zeolite coatings by the incorporation of zeolite powders in a sol-gel matrix, however, in these studies the corrosion resistance of the zeolite coated aluminium alloys are marginally higher than the only sol-gel matrix coated alloy. Kataria *et al.* [14] pressed zeolite powders on an adhesive coated mild steel and examined the corrosion resistance of the coated alloy in presence of various organic acids, such as, acetic acid, formic acid and citric acid. They concluded that presence of the zeolite coating plays an important role in preventing aggressive H^+^ ions from attacking the mild steel substrates, and hence, provides improvement in the corrosion resistance of the alloy. Cai *et al.* [11] have reported that zeolite coating developed on AA2024-T3 aluminium alloy via ionothermal synthesis provided excellent barrier properties to the alloy in 0.1 M NaCl solution. Most investigations on the barrier properties of zeolite coatings have been undertaken on aluminium alloys and steel [8,9,10,11,12,13,14]. There is only one report of the influence of zeolite coating on the corrosion resistance of a magnesium alloy (Mg-Li alloy) [15]. The zeolite coating developed on Mg-Li alloy was synthesized by hot pressing method using two different SDAs, tetrapropylammonium bromide (TPABr) and tetrapropylammonium hydroxide (TPAOH). Although the corrosion resistance of the zeolite coating containing TPABr was about on order of magnitude higher than the uncoated alloy, the zeolite coating containing TPAOH did not improve the corrosion resistance of the Mg-Li alloy in 3.5% NaCl solution.

In the present study, we have developed a zeolite coating with TPAOH as the SDA on aluminium containing magnesium alloy, AZ91D. To synthesize the zeolite coating on AZ91D, we have employed *in situ* crystallization technique (that has been reported to produce uniform coatings on aluminium alloys and steel [8,9,10]). The morphology of the coated and uncoated AZ91D was examined using SEM and the chemical nature of the zeolite coating was investigated by XRD. The corrosion resistance of the coated and the uncoated AZ91D was investigated using potentiodynamic polarization and electrochemical impedance spectroscopy (EIS) in 0.1 M NaCl solution. In order to examine the long-term durability of corrosion protection due to the coating, EIS was performed after different durations of immersion. An electrical equivalent circuit (EEC) has been identified to simulate the experimental impedance data and analyze the time dependent electrochemical evolutions at the zeolite coating/electrolyte and metal/electrolyte interfaces during immersion in 0.1 M NaCl solution.

## 2. Results and Discussion

### 2.1. Morphological Characterization

**Figure 1:**
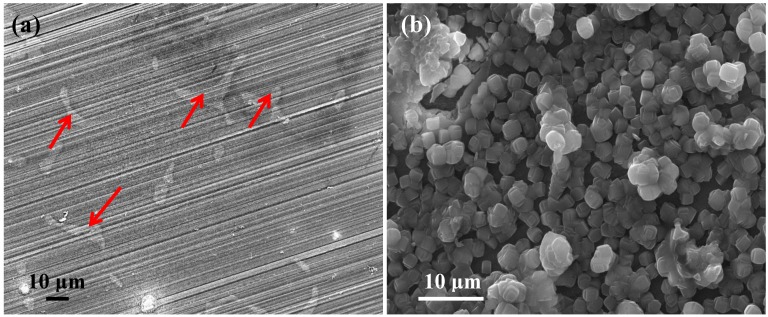
Scanning electron micrograph of (**a**) uncoated and (**b**) zeolite coated AZ91D magnesium alloy.

The surface morphology of the uncoated and zeolite coated AZ91D alloy is shown in Figure 1. The intermetallic particles (generally Mg_17_Al_12_ [16]) are clearly visible (shown by arrows in Figure 1a) along with the scratch marks of the uncoated alloy (from the 2500 grit SiC papers). The zeolite coated alloy was uniformly covered with cuboidal zeolite crystals with a typical size of ~2.5 μm.

### 2.2. Crystallography and Chemical Characterization

**Figure 2:**
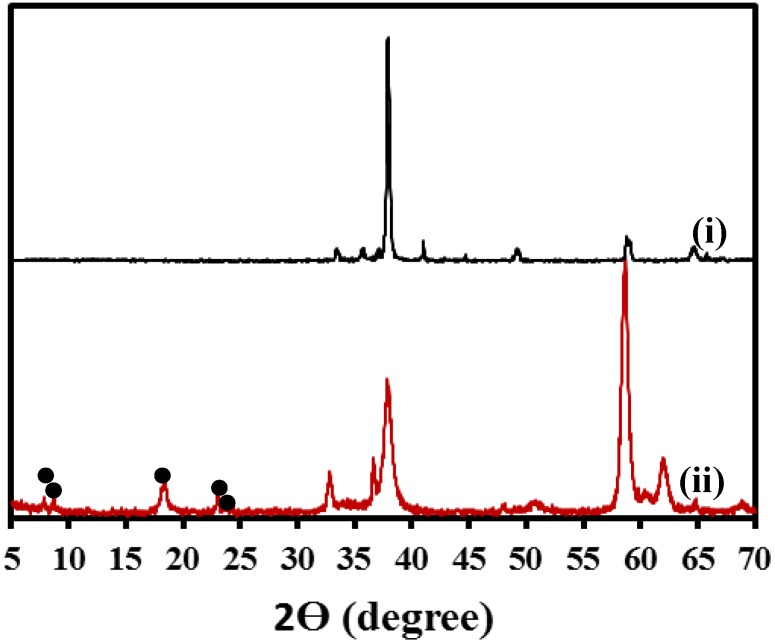
XRD spectra of (i) uncoated and (ii) zeolite coated specimens.

Crystalline nature of zeolite on the coated specimen was further confirmed by X-ray diffraction (XRD) analysis (Figure 2). The major diffraction peaks corresponding to zeolite crystals are indicated by the filled circles in Figure 2 and are consistent with the standard card PDF#37-0361 for the ZSM 5 zeolite structure (Na*_n_*Al*_n_*Si_96−*n*_O_192_·16H_2_O, where 0 < *n* < 27). Diffraction peaks associated with the uncoated AZ91D alloy was also present in the zeolite coated specimen. This may be attributed to the fact that the penetration depth of the X-rays was larger than the coating thickness, allowing signal from the alloy substrate underneath the coating. This observation is consistent with the XRD spectra reported for other zeolite coated magnesium alloy [15].

### 2.3. Electrochemical Characterization

**Figure 3:**
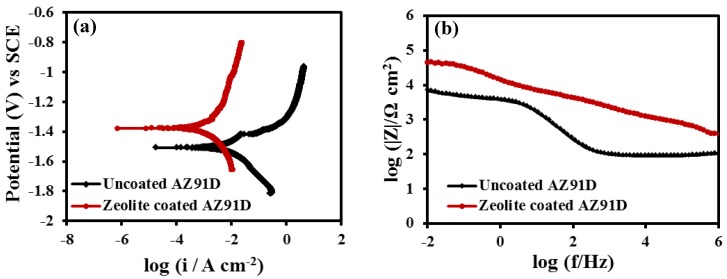
(**a**) Potentiodynamic polarisation and (**b**) Bode (|Z|) plots of the uncoated and zeolite coated AZ91D alloy at 2 h of immersion in 0.1 M NaCl solution.

Potentiodynamic polarisation plots of the zeolite coated and uncoated specimens after 2 h of immersion in 0.1 M NaCl solution are shown in Figure 3a. The corrosion potential (*E*_corr_), which is a measure of the corrosion susceptibility [17], of the zeolite coated specimen was 130 mV more positive as compared to the uncoated specimen. The anodic current density of the coated specimen was at least one order of magnitude lower than the uncoated specimen. The polarisation plots also indicated an observable suppression of pitting due to the zeolite coating. The significant positive shift (130 mV) of the *E*_corr_ and the substantial decrease in the anodic current density in case of the zeolite coated specimen can be attributed to the enhanced corrosion resistance due to the deposition of a uniform zeolite coating as shown in Figure 1b. Consistent with the polarization data, the magnitude of impedance, which is a measure of the corrosion resistance, is an order of magnitude higher for the zeolite coated specimen than the uncoated specimen (Figure 3b). This improvement is higher than that achieved due to a similar coating in the case of the Mg-Li alloy [15].

**Figure 4:**
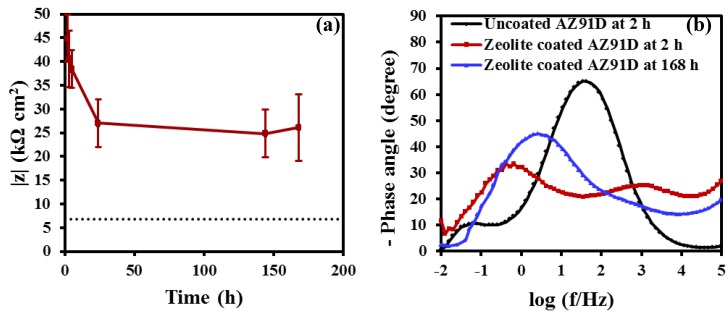
(**a**) Magnitude of impedance of the zeolite coated specimen at different durations of immersion in 0.1 M NaCl solution. The dashed line represents the magnitude of impedance of the uncoated specimen at 2 h of immersion in 0.1 M NaCl solution, and (**b**) phase angle plots of the uncoated and zeolite coated AZ91D specimens at various durations of immersion in 0.1 M NaCl solution.

Figure 4a shows the magnitude of impedance, *i.e.*, the corrosion resistance of the zeolite coated specimen at different durations of immersion in 0.1 M NaCl solution. For reference, corrosion resistance of the uncoated specimen at 2 h of immersion is also shown by the dotted line in Figure 4a. The corrosion resistance of the coated specimen decreased linearly in the first 24 h of immersion (from 45 kΩ·cm^2^ at 2 h to 27 kΩ·cm^2^ at 24 h), and then remained almost constant for the rest of the immersion test. It is noted that even after 168 h of immersion, the corrosion resistance of the coated specimen was at least five times higher than that of the uncoated specimen.

The phase angle plot of the zeolite coated specimen at 2 h of immersion in 0.1 M NaCl solution is characterized by two distinct time constants, a high frequency time constant, which is generally attributed to the electrochemical response at the coating/electrolyte interface [18,19,20,21,22,23], and, a low frequency time constant, which is commonly attributed to the diffusion of electrolyte and electrochemical response of the metal/electrolyte interface [19]. The high frequency time constant (representative of the coating/electrolyte interface) diminished at 168 h, and a single broad peak appeared in the low frequency range. This may be attributed to the overlap of two time constants for the responses of the coating/electrolyte and the metal/electrolyte interfaces [19]. The phase angle plot of the uncoated specimen is characterized by a time constant in the medium frequency range and a small time constant in the low frequency range. These time constants can be associated with the metal hydroxide/electrolyte and metal/electrolyte interfaces. The phase angle response of the uncoated specimen is consistent with those reported in the literature [24]. The diminution of the high frequency time constant of the zeolite coated specimen (Figure 4b) at 168 h of immersion is consistent with the trend of corrosion resistance (*i.e.*, a decrease of the corrosion resistance from 45 kΩ·cm^2^ at 2 h to 26 kΩ·cm^2^ at 168 h) offered by the zeolite coated specimen.

**Figure 5:**
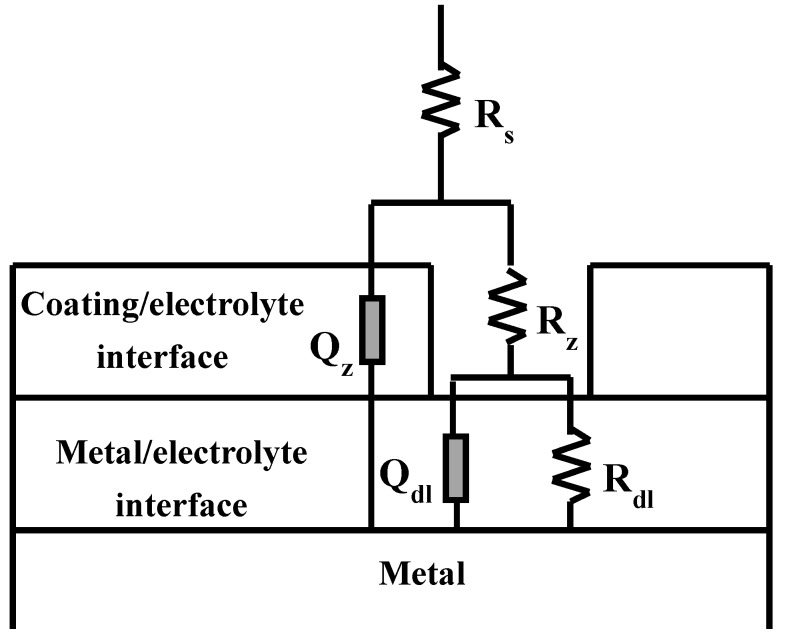
The electrical equivalent circuit (EEC) fitted to the experimentally obtained impedance data of the zeolite coated AZ91D alloy.

In order to understand the kinetics of the electrochemical phenomena at the different interfaces (coating/electrolyte and metal/electrolyte) and their contributions to the overall durability of the zeolite coating, an electrical equivalent circuit (EEC) has been used to analyse the impedance data. In the present study, we have used complex nonlinear least squares (CNLS) fitting to analyse the impedance data. The details of the CNLS method and the fitting procedure are described in the literature [25]. The circuit description code (CDC) used in this study is well established [26]. The impedance of the zeolite coated specimen was analysed by an EEC of description *R*_s_(*Q*_z_ [*R*_z_(*Q*_dl_*R*_dl_)]), shown in Figure 5, where the metal/electrolyte interface and the coating/electrolyte interface behave as a set of constant phase elements (CPEs) and resistances in parallel. Several other researchers have extensively used this particular EEC, in order to simulate the impedance data of the coated metals and alloy systems [19,20,21,27,28,29,30,31,32]. Chi square values in the range of 10^−4^ and the total error of less than 4% further confirmed that this EEC was a good fit to the impedance data of the zeolite coated specimens at different durations of immersion in 0.1 M NaCl solution.

**Figure 6:**
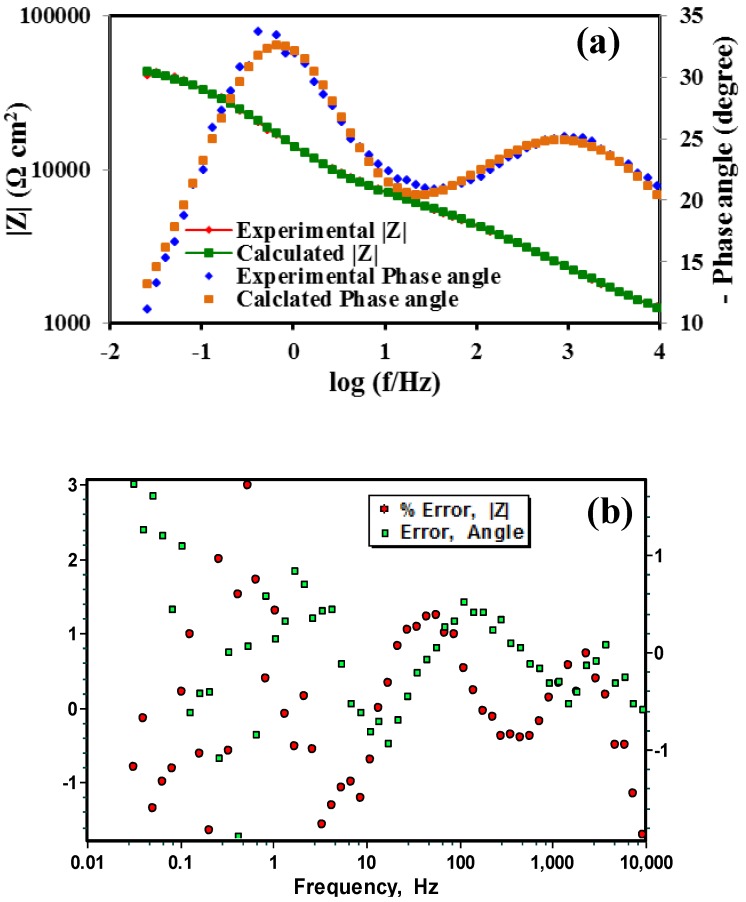
(**a**) Bode plots for the measured and the calculated values and (**b**) error plots of the zeolite coated AZ91D at 2 h of immersion in 0.1 M NaCl solution.

In this EEC (Figure 5), the electrolyte resistance is represented by *R*_s_. The coating/electrolyte interface is characterized by a CPE (*Q*_z_) and a pore resistance (*R*_z_). The other time constant present in the EEC is represented by the parallel combination of another CPE (*Q*_dl_) and a resistance, *R*_dl_ describing the electrochemical response of the metal/electrolyte interface. The incorporation of the CPEs in the proposed model provided better agreement between the simulated data and the experimental impedance data. The use of the CPE is justified by noting the distributed surface reactivity, roughness, electrode porosity, current and potential distributions associated with the electrode geometry and anion adsorption kinetics [33]. The simulated impedance data were in good agreement with the experimentally obtained data set for the coated specimens at all stages of immersion. Representative simulated and the observed Bode impedance plots of the zeolite coated specimens at 2 h of immersion in 0.1 M NaCl solution are shown in Figure 6a. Error plots for the coated specimens at different stages of immersion show that the maximum error in the simulated data are less than 3% for the modulus of impedance (|Z|) and are less than 2% for the phase angle. A representative error plot is shown in Figure 6b.

**Figure 7:**
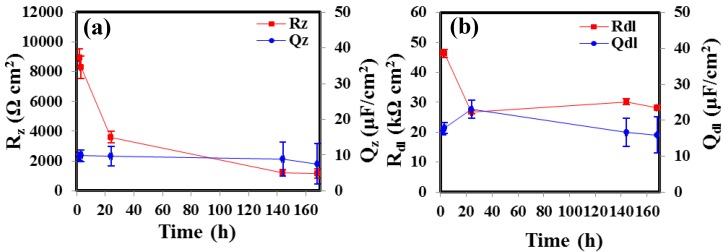
Temporal evolution of the different parameters associated with the proposed EEC for the zeolite coated AZ91D alloy at different durations of immersion in 0.1 M NaCl solution.

The time evolution of the EEC parameters associated with the coating/electrolyte interface and the metal/electrolyte interface of the zeolite coated AZ91D specimen is shown in Figure 7. The coating resistance, *R*_z_ decreased with time, whereas, *Q*_z_ remained constant (Figure 7a). Similar trend was also observed in case of the metal/electrolyte interface, where, *R*_dl_ decreases steadily till 24 h and then remain constant till 168 h, whereas, no appreciable change in *Q*_dl_ was observed over the entire duration. The decreases in *R*_z_ and *R*_dl_ over time are in agreement with the experimentally observed behaviour of the zeolite coated AZ91D specimen (Figure 4a), where the corrosion resistance decreases steadily till 24 h and then remain constant till 168 h.

### 2.4. Post corrosion Morphology

**Figure 8:**
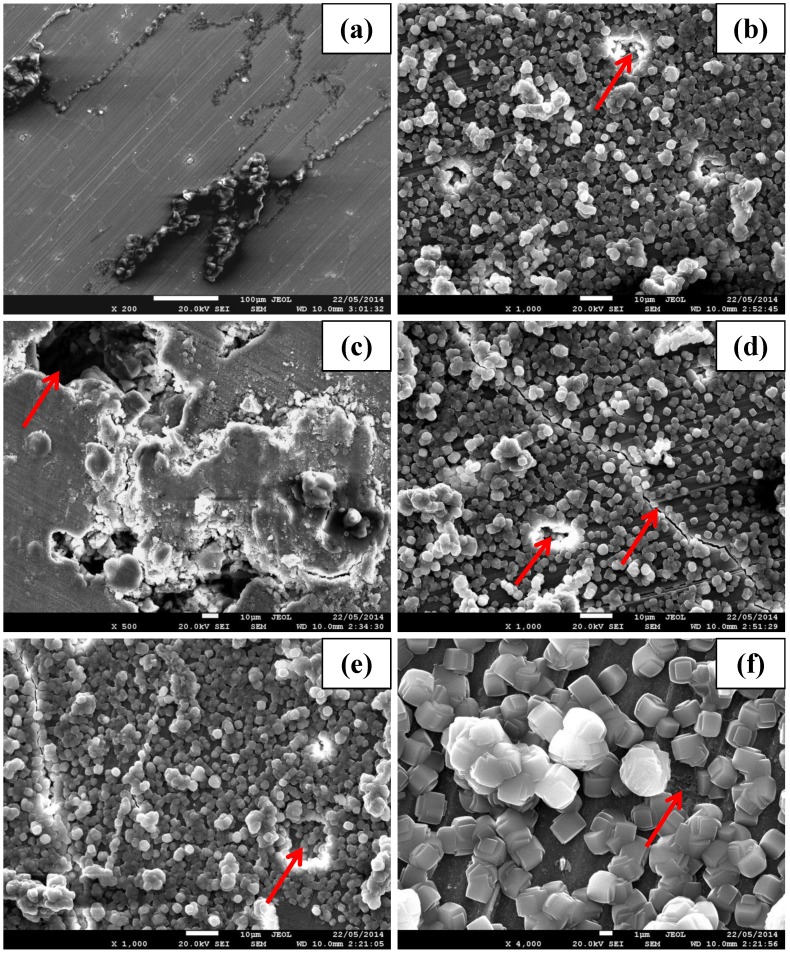
Scanning electron micrograph of the post corrosion morphologies of (**a**) uncoated and (**b**) zeolite coated specimens at 2 h of immersion. Corrosion morphologies of the (**c**) uncoated and (**d**) zeolite coated alloy at 24 h of immersion. Corrosion morphologies of (**e**) zeolite coated specimen at 168 h of immersion in 0.1 M NaCl solution and (**f**) magnified view of (**e**).

The corrosion morphologies of the zeolite coated and uncoated specimens are shown in Figure 8. At 2 h, thick corrosion products were scattered on the uncoated specimen (Figure 8a), whereas the zeolite coated specimen showed minimal degradation (indicated by a red arrow in Figure 8b), which is consistent with the EIS results for this specimen (that suggested higher corrosion resistance (45 kΩ·cm^2^)). After 24 h, besides the large amount of thick corrosion products, large pits were also observed over the uncoated specimen surface (Figure 8c). During this exposure, although the coated specimen was still covered primarily with the zeolite coating, the coating had developed large cracks (indicated by an arrow in Figure 8d), which explains the decrease in the corrosion resistance of this specimen to 27 kΩ·cm^2^ at 24 h (from 45 kΩ·cm^2^ at 2 h). However, the surface morphology of the coated specimen did not suffer further deterioration during further immersion till 168 h (Figure 8e), and hence, the corrosion resistance after 168 h were similar to that at immersions for 24 h (Figure 4a). A high magnification micrograph of the zeolite coated specimen at 168 h (Figure 8f) indicated the onset of corrosion underneath the coating (as shown by an arrow in Figure 8f). This is in agreement with the decrease in *R*_z_ and *R*_dl_.

The electrochemical results (potentiodynamic polarisation and EIS) suggest that zeolite coating significantly increases (at least one order of magnitude) the corrosion resistance of the AZ91D alloy. This improvement can be attributed to the development of a uniform and inert zeolite coating on the alloy surface (as is evident in Figure 1b). The coating resistance decreases steadily in the first 24 h of immersion due to the development of cracks, but remains largely unaltered during subsequent immersion until 168 h. At 168 h immersion the corrosion resistance of the zeolite coated specimen is at least five times higher than the uncoated specimen (Figure 4a). This improvement in corrosion resistance due to zeolite coating developed on AZ91D is consistent with the reports of the corrosion resistance due to zeolite coating in case of aluminium alloys and steel [8,9,10,11,12,13,14], but is higher than that reported for a similar zeolite coating (with TPAOH as the SDA) developed on another magnesium alloy (Mg-Li alloy) [15].

## 3. Experimental Procedure

### 3.1. Material Used

Rectangular specimens (13 mm × 13 mm × 9 mm) of as-cast AZ91D alloy (consisting of 9% Al, 1% Zn) were machined from the ingot, and ground up to 2500 grit silicon carbide (SiC) paper. The samples, duly rinsed with acetone and dried using compressed air, were subjected to zeolite coating, chemical and electrochemical analysis.

### 3.2. Synthesis of the Coating Solution

Zeolite coating solution was prepared using in-situ hydrothermal crystallization method reported by Cheng *et al.* [8] 5.36 g of sodium hydroxide (NaOH) was added to 336 mL of deionized (DI) water. 0.0105 g of aluminium foil was then added to the sodium hydroxide solution and was left to stir for about 10 min in order to fully dissolve the aluminium foil. This was followed by drop-wise addition of 35 mL of tetraproylammonium hydroxide (TPAOH) and 45 mL of tetraethylorthosilicate (TEOS). The clear solution was stirred overnight at room temperature.

### 3.3. Deposition of the Coating

A Teflon coated autoclave was used as the coating synthesis vessel. AZ91D specimens were placed horizontally in the vessel and were completely immersed in the clear coating solution. Crystallization of the coating was carried out at 175 °C for 5 h. After the crystallization process, the autoclave was removed from the oven and was water quenched. The coated specimens were rinsed with DI water and were dried using compressed air.

### 3.4. XRD Analysis

The coating was analyzed by X-ray diffraction (XRD) using a Phillips 1140 diffractometer (Eindhoven, The Netherlands) with Cu K_α_ line generated at 40 kV and 25 mA at a scan rate of 2°/min and a step size of 0.02°.

### 3.5. SEM Analysis

The morphology of the zeolite coated and uncoated specimens before and after corrosion was analysed using a JEOL JSM 7001 FEG SEM (Tokyo, Japan). The coated and the corroded specimens were gold coated to avoid charging.

### 3.6. Electrochemical Characterization

Potentiodynamic polarization and electrochemical impedance spectroscopy (EIS) were performed in 0.1 M sodium chloride (NaCl) solution using a Biologic VMP3 potentiostat and an electrochemical cell with three electrodes (specimens with an exposed area of 0.785 cm^2^ acted as the working electrode, platinum mesh as counter electrode and saturated calomel electrode as the reference electrode). All the experiments were performed at room temperature. Open circuit potential (OCP) was monitored for 1 h to confirm its stability with time. A fluctuation of OCP within 10 mV for a period of 1000 s was considered as a stable potential before carrying out the corrosion tests. Potentiodynamic polarization tests were carried out at a scan rate of 0.5 mV/s starting at −250 mV with respect to the OCP. EIS was performed on all the specimens after 1 h of immersion in 0.1 M NaCl solution. The impedance tests were carried out by applying a sinusoidal potential wave at OCP with an amplitude of 10 mV. Impedance response was measured over frequencies between 1 MHz and 10 mHz, recording 10 points per decade of frequency. Impedance analysis was carried out using PAR ZSimpWin package for Windows generally for frequencies between 100 kHz and 50 mHz to prevent misinterpretation of any artefacts that may be present in high frequency region, or the scatter in low frequency region. All the electrochemical tests were repeated at least thrice to examine the reproducibility of the results.

## 4. Conclusions

*In situ* crystallization produced uniform zeolite coating on AZ91D magnesium alloy. The corrosion resistance of the zeolite coated specimen was at least one order of magnitude higher than the uncoated specimen at the initial stages of immersion. Even after 168 h of immersion in 0.1 M NaCl solution the corrosion resistance of the zeolite coated specimen is at least five fold higher than the uncoated specimen. The improvement in the corrosion resistance of AZ91D alloy due to the application of zeolite coating can be attributed to the uniformity and the inert nature of this coating.
